# Clinical Utility of Amplification Refractory Mutation System-Based PCR and Mutation-Specific PCR for Precise and Rapid Genotyping of Angiotensin-Converting Enzyme 1 (ACE1-rs4646996 D>I) and Angiotensin-Converting Enzyme 2 (ACE2-rs4240157T>C) Gene Variations in Coronary Artery Disease and Their Strong Association with Its Disease Susceptibility and Progression

**DOI:** 10.3390/diagnostics12061321

**Published:** 2022-05-26

**Authors:** Aadil Yousif, Rashid Mir, Jamsheed Javid, Jameel Barnawi, Mohammed M. Jalal, Malik A. Altayar, Salem Owaid Albalawi, Faisel M. Abuduhier

**Affiliations:** 1 Department of Medical Laboratory Technology, Faculty of Applied Medical Sciences, University of Tabuk, Tabuk 71491, Saudi Arabia; mjalal@ut.edu.sa (M.M.J.); maltayar@ut.edu.sa (M.A.A.); 2 Prince Fahd Bin Sultan Research Chair, Department of Medical Lab Technology, Faculty of Applied Medical Sciences, University of Tabuk, Tabuk 71491, Saudi Arabia; drjamsheedjavid@gmail.com (J.J.); jbarnawi@ut.edu.sa (J.B.); fabu-duhier@ut.edu.sa (F.M.A.); 3 Department of Cardiology, King Fahd Speciality Hospital, Tabuk 71491, Saudi Arabia; sal-wabsy@moh.gov.sa.com

**Keywords:** coronary artery disease (CAD), angiotensin l-converting enzyme (ACE), insertion/deletion (I/D), T-ARMS—Tetra Primer-Amplification Refractory Mutation System, MS-PCR-mutation specific PCR, Hardy–Weinberg disequilibrium (HWD)

## Abstract

**Background:** Experimental clinical and research studies demonstrated that the renin–angiotensin system (RAS) affects the pathogenesis of atherosclerosis and the prognosis of coronary heart disease (CHD). The results show that ACE2 (angiotensin I-converting enzyme 2) might act as a protective protein for cardiovascular diseases; however, only a few studies in human populations have been carried out. The aim of this study was to develop, optimize, and validate a direct T-ARMS-based PCR assay for the precise and rapid genotyping of ACE1-rs4646996 D>I and ACE2-rs4240157T>C and study their association with coronary artery disease susceptibility and progression. **Methodology:** This study included 149 consecutive coronary artery disease patients and 150 healthy controls. We utilized T-ARMS for the precise and rapid genotyping of ACE2-rs4240157; rs4646994. **Results:** Our results indicated that the ACE1-rs4646996 D>I genotypes observed between CAD cases and controls were statistically significant (*p* < 0.008) and, similarly, the ACE2-rs4240157T>C genotypes observed were significant (*p* < 0.0001). Moreover, the frequency of the D allele (ACE1-D>I) and C allele (ACE2-rs4240157T>C) was found to be higher among CAD patients than the HC. Our results indicated that in the codominant model, the ACE2-ID genotype was strongly associated with increased CAD susceptibility in a codominant model with an OR of 2.37, (95%) CI = (1.023–5.504), and *p* < 0.04. Similarly, the ACE2-DD genotype was strongly associated with an increased CAD susceptibility with an OR of 3.48, (95%) CI = (1.49 to 8.117), and *p* < 0.003. Similarly, in allelic comparison, the D allele was strongly associated with CAD susceptibility with an OR of 1.59, (95%) CI = (1.12–2.24), and *p* < 0.003. Our results revealed that there was a significant correlation between ACE2-I/D genotypes and hypertension, T2D, and obesity (*p* < 0.05). The results of ACE2 rs4240157 genotyping indicated a strong association in the codominant model with an increased CAD susceptibility with an OR of 3.62, (95%) CI = (2.027 to 6.481), and *p* < 0.0001. Similarly, in a dominant inheritance model, a strong association is observed between the ACE2 rs4240157 (CT+CC) genotype with an OR of 6.34, (95%) CI = (3.741 to 10.749), and *p* < 0.0001. In allelic comparison, the T allele was strongly associated with CAD susceptibility with an OR of 5.56, (95% CI = (3.56 to 7.17), and *p* < 0.0001. Similarly, our results revealed that there was a significant association of the ACE2-rs4240157T>C genotypes with Triglycerides (mg/dL), HDL-C (mg/dL), total Cholesterol (mg/dL), and C-reactive protein (mg/L) in CAD. **Conclusion:** It was indicated that the ARMS technique and MS-PCR assay proved to be fast, accurate, and reliable for ACE2-rs4240157T>C and ACE1-rs4646996 D>I, respectively, and can be used as a potential molecular tool in the diagnosis of genetic diseases in undeveloped and developing countries—where there might be a shortage of medical resources and supplies. ACE1-I>D genotypes were strongly associated with T2D, hypertension, and obesity (*p* < 0.002). Besides the ACE2-rs4240157 CT heterozygosity genotype, the T allele was strongly associated with CAD susceptibility. Future longitudinal studies in different ethnic populations with larger sample sizes are recommended to validate these findings

## 1. Introduction

Coronary artery disease (CAD), also known as atherosclerotic heart disease, is the first cause of morbidity and mortality in developed countries all over the world [1]. During the last 30 years, important progress was made in order to diminish the morbidity and mortality of atherosclerotic disease. [2] In Saudi Arabia, the all prevalence of CAD is 5.5% and it is considered a major cause of death among adults over the age of 35 years. In KSA, the association of CAD with a higher mortality rate in Saudi patients with the prevalence of risk factors is more significant than in the Western population [3,4]. Since the 1960s they have known that myocardial infarction and all atherosclerotic disease has an important hereditary component [5], and CAD is a polygenic disorder with multiple genetic variants. [6] In addition, the exact pathogenesis of coronary artery disease is unknown [7], but some studies reported that CAD is a complex chronic inflammatory disease, characterized by the straightening of the coronary arteries that supply oxygen to the heart [8]. CVD represents a paradigm for the complex interplay between environmental risk factors and is a multifactorial disease affected by natural and hereditary elements [9]. Besides the family history of premature CAD, there are other hazard components, such as smoking, obesity, diabetes, hypertension, and dyslipidemia—all these interactive factors contribute to the occurrence of the disease [10,11].

### Angiotensin Converting Enzyme-2 Gene System

There was a strong association between angiotensin-converting enzyme (ACE) gene polymorphism and several cardiovascular diseases, such as myocardial infarction, left ventricular hypertrophy, and restenosis after percutaneous transluminal coronary angioplasty [12,13]. The renin–angiotensin system (RAS) plays a central role in the control of cardiovascular and renal functions by maintaining homeostasis of blood pressure and electrolyte balance [14]. The angiotensin-converting enzyme polymorphism gene had revealed a strong association with ACE activity, premature atherosclerosis, and myocardial infarction [15,16]. ACE converts the inactive angiotensin I into the vasoactive and aldosterone-stimulating octapeptide angiotensin II, as well as causing the inhibition of bradykinin. For these reasons, the ACE gene has been a preferred target in unraveling the molecular complex structure of cardiovascular diseases [17,18]. It was found that the ACE insertion/deletion (I/D) gene polymorphism (rs4340) is a 287 bp sequence of DNA in intron 16 of the ACE gene on chromosome 17q23, whereas there are a few polymorphisms that are in an unbalanced linkage at (rs4646994) in some populations [19,20]. The association between ACE I/D (rs4340) and 2350A>G (rs4343) polymorphisms has been found in several studies, including ones that investigated high blood pressure, systemic lupus erythematous, diabetic nephropathy, Alzheimer’s disease, and renal diseases. According to age, in adults, plasma ACE does not change and is only influenced by environmental or lifestyle factors—to a lesser extent [21]. Nowadays, different molecular assays have been utilized to detect genetic mutations, like Sanger sequencing, but this technique cannot rapidly screen large numbers of mutations in samples. Fortunately, this problem can be solved by massive next-generation sequencing (NGS) [22]. However, both Sanger sequencing and NGS are expensive. ARMS-PCR is based on the principle that the 3′-terminal nucleotides of the PCR primer must be complementary to its target sequence for efficient amplification. Amplification refractory mutation system PCR (ARMS-PCR) has been approved by the China Food and Drug Administration (CFDA) and has become a widely used method in clinical practice [23,24]. ARMS-PCR is more sensitive than Sanger sequencing due to its detection limit being about 1% [25]. The ARMS technique demands only PCR amplification and gel electrophoresis of the amplicons. To the best of our knowledge, there is no report that has investigated ACE2 gene polymorphisms in CAD patients in Tabuk. The aim of this study was to develop, optimize, and validate a direct T-ARMS-based PCR assay for the precise and rapid genotyping of ACE2 (rs4240157, rs4646994) and study their association with coronary artery disease susceptibility and progression.

## 2. Methodology

### 2.1. Study Population

This study was a population-based cohort study designed to investigate risk factors for CAD related to outcomes in middle-aged men and women from India. A total of 200 blood samples were analyzed, among which 149 were from CAD patients (100 men and 49 women) and 150 were healthy controls.

### 2.2. Ethics Approvals

The ethical approvals were obtained from the local ethics committee from the University of Tabuk (Decision No:UT-91-23-2020) and were in accordance with the standards for human subjects outlined in the principles of the Helsinki Declaration. Informed consent was obtained before collecting samples from all patients and control subjects.

### 2.3. Patient Selection Criteria Inclusion Criteria

Patients undergoing elective angiography for the evaluation of stable chest pain at the King Fahd Special Hospital, Saudi Arabia were recruited. Some non-invasive tests were performed, including an electrocardiogram (ECG or EKG), ambulatory electrocardiography, Holter monitoring, chest X-ray, echocardiogram (echo), cardiac computed tomography (CCT), exercise stress test, and Myocardial Perfusion Imaging (MPI) or Multigated acquisition scan (MUGA). The cohort was classified based on their coronary angiographic findings as either significant CAD (stenosis ≥ 50%) or ICAD (no stenosis or stenosis < 50%).

### 2.4. Exclusion Criteria

The exclusion criteria included patients with a history of non-coronary cardiac disorders cases, previously performed coronary bypass surgery, or percutaneous transluminal coronary angioplasty (PTCA) due to their treated coronary status.

### 2.5. Selection Criteria of Healthy Controls

A healthy control cohort was established from participants visiting for routine checkup to King Fahd Special Hospital, Saudi Arabia. These participants completed the informed consent form and questionnaire. The healthy control cohort was selected based on the self-reported absence of previous heart attack or angina. Some biochemistry tests were also performed. The exclusion criteria included participants with available cardiac history, history of angina, and/or myocardial infarction.

### 2.6. Measurement of Serum Lipids

After overnight fasting and prior to coronary angiography, blood was collected from each subject. Serum total cholesterol, triglyceride, high-density lipoprotein (HDL) and low-density lipoprotein (LDL)-C concentrations, and total cholesterol/HDL-C ratios were determined using the standard method.

### 2.7. Data Collection

The corresponding data from each patient were collected and analyzed for their cholesterol level, diabetes, RBS, LDL, HDL, TGL, and smoking. Patient follow-up was performed regularly, and samples were collected on a regular basis.

### 2.8. Sample Collection from CAD Patients

About 3 mL of peripheral blood samples were collected in an EDTA or Lavender top tube for all CAD patients. The blood specimens were immediately stored at −20 °C until use.

### 2.9. Sample Collection from Healthy Controls

All healthy, age-matched control specimens were timed around routine blood draws that are part of a routine workout, and hence did not require additional phlebotomy. All participants were provided written informed consent forms. About 3 mL of peripheral blood was collected in EDTA tubes. The blood specimens were immediately stored at −20 to −30 °C.

### 2.10. Genomic DNA Extraction

Genomic DNA was extracted using the DNeasy Blood Kit (Qiagen, Hilden, Germany) per the manufacturer’s instructions. The extracted DNA was dissolved in nuclease-free water and stored at 4 °C until use. The quality and integrity of the DNA were checked by NanoDrop™ (Thermo Scientific, Waltham, MA, USA). All DNA samples from the CAD and control patients were screened for purity by measuring the optical density (OD) at A260 and A280. The A_260_/A_280_ ratios ranged from 1.80–1.96, indicating good quality DNA.

### 2.11. Genotyping of ACE 1 and ACE 2 Genes

Initially, the T-ARMS-PCR primers of the ACE2 rs4240157T>C gene polymorphism and ACE-1-rs4646994 I/D were designed using online free software (http://cedar.genetics.soton.ac.uk (accessed on 25 March 2022)) and are depicted in Table 1. After encountering some problems with the T-ARMS-PCR for the ACE -1(rs4646996 (D>I) gene polymorphism, we then designed two mutation-specific primers for the ACE-1-rs4646994 I/D polymorphism, as depicted in Table 1.

### 2.12. Optimisation of T-ARMS-PCR Primers for ACE-1-rs4646994 I/D Gene Polymorphism

The inner primers were positioned asymmetrically for the common (outer) primers to generate allele-specific amplicons with different product lengths. Considering the importance of melting temperature (Tm) on the sensitivity of T-ARMS-PCR, the inner primers were modified to make the Tm close to its counter primer (forward-reverse). The criterion set was that the Tm difference should be less than or equal to 2 °C between primer combinations that make PCR amplicons in a reaction, i.e., OF/OR, IF/OR, IR/OF (Table 1). The primer specificity was tested using the BLAST program of NCBI.

### 2.13. Preparation of the PCR Cocktail

T-ARMS-PCR was performed with a reaction volume of 25 µL containing template DNA (50 ng); 0.25 µL of primer stock solution of FO, RO, FI, and RI, which containing 5 pmol of each primer; and 10 µL from GoTaq^®^ Green Master Mix (cat no M7122); (Promega, Madison, WI, USA). The final volume of 25 µL was adjusted by adding nuclease-free ddH_2_O. Finally, 2 µL of DNA was added from each subject.

### 2.14. Thermocycling Conditions

Touchdown PCR amplification (Touchdown) was performed with an initial denaturation at 95 °C for 2 min, followed by a denaturation at 95 °C for 25 s with a first annealing at 69 °C to 60 °C (14 cycles), and the remaining cycles (28 cycles) were performed with an annealing at 60.5 °C for 55 s and extension at 72 °C for 55 s, which was followed by a final extension for 10 min. The amplified products were run on non-denaturing polyacrylamide gel electrophoresis (6%) and were stained with silver staining method.

### 2.15. Gel Electrophoresis

The ACE 1-rs4646996 D>I polymorphisms are located on intron 16 with a 139 bp distance. Four primers were used in the same PCR. Two primers, Fo and Ro, were designed to amplify a 712 bp band (I allele) and 424 bp band (D allele), which serve as control bands for the success of the amplification. Two specific primers, inner forward FI and inner reverse RI for rs4343 with complementary 3′-end nucleotides and corresponding polymorphisms, were introduced. We encountered some problems with the T-ARMS-PCR primers for the *ACE* 1-rs4646996D>I genotyping. We designed two mutation-specific primers for ACE1-rs4646996 D>I genotyping. After optimization mutation-specific PCR worked perfectly for *ACE* 1-rs4646996D>I genotyping therefore we discontinued ARMS-PCR. 

### 2.16. Optimization of Mutation Specific PCR for ACE 1-rs4646994 I>D Polymorphism

**Figure 1:**
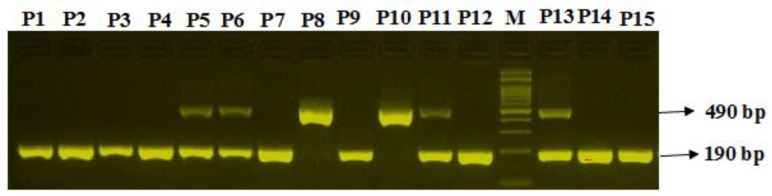
Optimization of Angiotensin-Converting Enzyme 2 genotyping ACE2-rs4646994 D/I in coronary artery disease patients. Legend: M-100 bp DNA ladder; Heterozygous D/I-P5,P6,P11,P13; Homozygous II-P8,P10; Homozygous DD-P1,P2,P3,P4,P7,P9,P12,P14 & P15.

The PCR was performed with a reaction volume of 12.5 µL and contained template DNA (50 ng), Fo—0.12 µL, R0—0.12 µL, RI—0.14 µL, and RI—0.14 µL of 10 pmol of each primer, as well as 6 µL from the GoTaq^®^ Green Master Mix (cat no M7122) (Promega Corp. Madison, WI, USA). The final volume of 12.5 µL was adjusted by adding nuclease-free double distilled water (ddH_2_O). Finally, 2 µL of DNA was added from each patient. The thermocycling conditions used were: 95 °C for 8 min, followed by 45 cycles at 94 °C for 30 s, an annealing temperature of 58 °C for 45 s and 72 °C for 50 s, followed by the final extension at 72 °C for 10 min. The PCR products were separated on a 2.5% agarose gel stained with 3 µL of sybre safe stain (Thermo Scientific, Waltham, MA, USA) and visualized on a UV trans illuminator from Bio-Rad (Hercules, CA, USA) to visualize three patterns: I/I (490-bp fragment), D/D (190-bp fragment), and I/D (both 490- and 190-bp fragments), as depicted in Figure 1.

### 2.17. Genotyping of ACE2 rs4240157T>C by ARMS-PCR

Tetra Primer-Amplification Refractory Mutation System based Polymerase Chain Reaction (T-ARMS PCR) can make DNA testing faster in a low-cost setting. The present study was aimed to design, optimize, and validate a T-ARMS PCR for faster genotyping of ACE2 rs4240157T>C.

#### 2.17.1. Preparation of PCR Cocktail 

T-ARMS-PCR was performed for the ACE2 rs4240157T>C gene polymorphism with a reaction volume of 12.5 µL that contains template DNA (50 ng); 0.12 µL of primer stock solution for FO, RO, FI, and RI, which contain 10 pmol of each primer; and 10 µL from GoTaq^®^ Green Master Mix (cat M7122); (Promega, Madison, WI, USA). The final volume of 12.5 µL was adjusted by adding nuclease-free ddH_2_O. Finally, 2.5 µL of DNA was added from each CAD subject.

#### 2.17.2. Gradient PCR and Thermocycling Conditions

Gradient PCR was performed for the ACE2 rs4240157T>C gene polymorphism using different annealing temperatures and a lesser number of cycles (25 to 35 cycles). This optimization was achieved in a series of optimization experiments in a single run by the gradient PCR machine from the thermocycler from Bio-Rad (Hercules, CA, USA)). The final annealing temperature was achieved at 60 °C at 30 cycles.

The final thermocycling conditions optimized for the ACE2 rs4240157T>C gene polymorphism were: initial denaturation temperature of 95 °C for 8 min, followed by 30 cycles at 94 °C for 30 s, an annealing temperature of 60 °C for 30 s and 72 °C for 35 s, followed by the final extension at 72 °C for 12 min, and storage at 4 °C until use.

#### 2.17.3. Gel Electrophoresis for ACE2 rs4240157T>C Amplification

**Figure 2:**
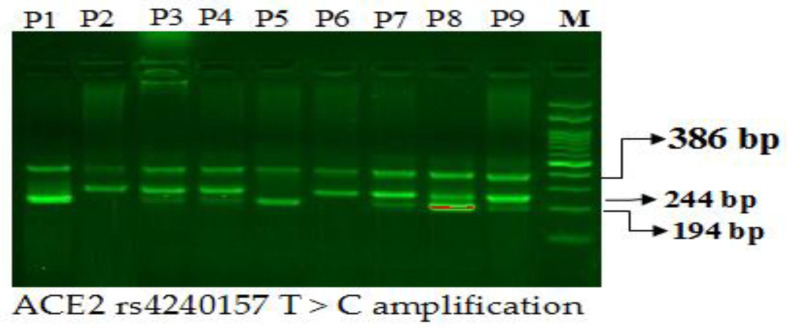
Optimization of Angiotensin-Converting Enzyme 2 genotyping ACE2 rs4240157T>C gene in coronary artery disease patients. Legend: M-100 bp DNA ladder; Heterozygous T/C-P3,P4,P7,P8 & P9; Homozygous CC-P1,P5; Homozygous TT-P2,P6.

Primers Fo and Ro flank the exon of the ACE2 gene, resulting in a band of 386 bp that act as a control for DNA quality and quantity. Primers FI and Ro amplify a wild-type allele (T allele), thereby generating a band of 244 bp, and primers Fo and RI generate a band of 194 bp from the mutant allele (C allele) as depicted in Figure 2.

#### 2.17.4. Statistical Analysis

The associations between the ACE-1-rs4646994 I>D and ACE2 rs4240157C>T genotypes and risk for CAD patients were estimated by computing the odds ratios (ORs), risk ratios (RRs), and risk differences (RDs) with 95% confidence intervals (CIs). Hardy–Weinberg disequilibrium (HWD) Deviations was calculated by chi-square (χ^2^) goodness-of-fit tests. Group differences were compared using Student’s two-sample t-test or one-way analysis of variance (ANOVA) for continuous variables and chi-squared for categorical variables. Allele frequencies among cases as well as controls were evaluated by using the chi-square Hardy–Weinberg equilibrium (HWE) test. A *p* value < 0.05 was considered significant. All statistical analyses were performed using SPSS 16.0. The univariate and multivariate analyses were calculated by using MedCalc software, version 20.027 (medcalc.org/calc/odds_ratio.php).

## 3. Results

### 3.1. Demographic Features

All the demographic features of the 150 consecutive CAD patients are summarized in Table 2. Of the 150 consecutive patients, 96 (64%) patients were below or equal to 50 years of age and 54 (36%) were above 50 years of age, A hundred and eight (72%) were males and 42 (28%) were females. Among 150 coronary artery disease patients, 76 (50.66%) had T2DM, 61 (40.66%) were having hypertension, 72 (48%) were obese, and 84 were having myocardial infarction.

### 3.2. Hardy–Weinberg Equilibrium

The genotype distributions and allele frequencies of the SNPs located in the ACE2-rs4646994 I/D showed no deviation in HWE (χ^2^ = 1.08 *p* < 0.29) in the control group HWE (χ^2^ = 0.008 *p* < 0.92). Thus, we chose 10% of the samples from the normal control group randomly to review the genotyping results, thereby showing that the accuracy rate was more than 99%.Similarly the frequency of the ACE2 rs4240157T>C gene polymorphism in control group showed no deviation in HWE (χ^2^ = 3.50 *p* < 0.06). Thus, we chose 10% of the samples from the normal control group randomly to review the genotyping results, thereby showing that the accuracy rate was more than 99%.

### 3.3. Statistical Comparisons between Patients and Controls (p Values) for ACE I/D Genotypes

The frequency of ACE2 genotypes in CAD cases and controls was II (6%), ID (43.33%) and DD (50%) and controls II (15.33%), and ID (46.6%) and DD (36.66%), respectively (Table 3). The ACE1-rs4646994 I/D gene variation observed between CAD patients and controls was statistically significant (*p* < 0.008). Moreover, the frequency of the D allele was found to be higher among CAD patients than in HC (0.72 vs. 0.60). (Table 3)

### 3.4. Multivariate Analysis of ACE2 I/D Polymorphism in the Coronary Artery Disease Patients and Healthy Controls

A multivariate analysis based on a logistic regression like an odds ratio (OD) or risk ratio (RR) with 95% confidence intervals (CI) were calculated for each group to estimate the association between the ACE2-rs4646994 I/D genotypes and risk of CAD—and the data are summarized in Table 4. Our results indicated that in the codominant model, the ACE2-ID genotype was strongly associated with increased CAD susceptibility with an OR of 2.37, (95%) CI = (1.023–5.504), RR = 1.38 (1.057–1.817), and *p* < 0.044. Similarly, the ACE2-DD genotype was strongly associated with an increased CAD susceptibility with an OR of 3.48, (95%) CI = (1.4961 to 8.1170), RR = 1.69 (1.26–2.282), and *p* < 0.003. There is a strong association observed between the ACE2–II and ACE2–(DI+DD) genotypes in the dominant inheritance model and leads to increased CAD susceptibility with an OR = 2.86, (95%) CI (1.27–6.41), RR= 1.52(1.1850 to 1.9593), and *p* < 0.010 (Table 4). There is a strong association observed between the ACE2–DD and ACE2–(II+DD) genotypes in the recessive inheritance model and leads to an increased CAD susceptibility with an OR = 1.71, (95%) CI (1.08–2.72), RR = 1.31 (1.033 to 1.676), and *p* < 0.022 (Table 4). In allelic comparison, the D allele was strongly associated with T2D susceptibility with an OR of 1.66, (95% CI) (1.1830 to 2.3557), RR 1.27(1.0906 to 1.5003), and *p* < 0.003.

### 3.5. Association of Biochemical and Clinical Features of Coronary Artery Disease Patients with ACE2 I/D Genotypes

**Association with gender and age**: Our results indicated that there was a significant association between ACE2-rs4646994 I/D genotypes and the age of the coronary artery disease patients (*p* < 0.022), Table 5). However, the higher incidence of heterozygosity was reported among coronary artery disease patients who were younger (less than 50 years), whereas the result showed that there is no association between ACE2-rs4646994 I/D genotypes and the gender of patients with a (*p* < 0.33).
**Association with Lipid profile biomarkers**: Association with total Cholesterol (mg/dL), as depicted in Table 5. The statistical analysis of the association between ACE2-rs4646994 I/D genotypes and Cholesterol (mg/dL) levels in the blood of coronary artery disease patients revealed a significant association (*p* < 0.002).
**Association with LDL-C (mg/dL)**: Moreover, the result showed that there is no significant association between ACE2-rs4646994 I/D genotypes and LDL-C (mg/dL) of coronary artery disease patients with a (*p* < 0.080).
**Association with HDL-C (mg/dL)**: Our result showed that there is a strong significant association between ACE2-rs4646994 I/D genotypes and HDL-C (mg/dL) of coronary artery disease patients with a (*p* < 0.035).
**Association with Triglycerides (mg/dL)**: A strong association was the association between ACE2-rs4646994 I/D genotypes and triglycerides (mg/dL) of coronary artery disease patients with a (*p* < 0.004).
**Association with hypertension and diabetes:** Our result revealed that there was a significant correlation between ACE2-rs4646994 I/D genotypes and hypertension (*p* < 0.002). Similarly, a significant correlation was reported between ACE2-rs4646994 I/D genotypes and diabetes (*p* < 0.0001).
**Association with Creatinine (mg/dL)**: The statistical analysis of the correlation between ACE2 I/D genotypes and Creatinine (mg/dL) in coronary artery disease patients revealed a non-significant association (*p* < 0.42).
**Association with C-reactive protein (mg/L)**: The statistical analysis of the correlation between ACE2 I/D genotypes and C-reactive protein (mg/L) in coronary artery disease patients revealed a significant association (*p* < 0.0001).
**Association with hypertension and diabetes:** Our result revealed that there was a significant correlation between ACE2 I/D genotypes and hypertension (*p* < 0.002) in coronary artery disease patients. Similarly, a significant correlation was reported between ACE2 I/D genotypes and diabetes (*p* < 0.0001) in coronary artery disease patients.
**Association with smoking and obesity**: Our result revealed that there was no significant correlation between ACE2 I/D genotypes and smoking (*p* < 0.74) in coronary artery disease patients. Similarly, a significant correlation was reported between ACE2 I/D genotypes and obesity (*p* < 0.040).
**Association with Myocardial infarction (MI)**: Our result revealed that there was a significant correlation between ACE2 I/D genotypes and myocardial infarction (MI) (*p* < 0.0001) in coronary artery disease patients.

### 3.6. Statistical Comparisons between CAD Patients and Controls (p Values) for ACE2 rs4240157T>C Genotypes

In CAD patients, the TT, TC, and CC genotype frequencies were 18%, 36%, and 46%, respectively, whereas in healthy controls the TT, TC, and CC genotype frequencies were 57.23%, 31.57%, and 9.86%, respectively (Table 6). The distribution of ACE2 rs4240157T>C genotypes observed between CAD patients and healthy controls was significant (*p* < 0.0001). Moreover, the frequency of the C allele (fC) was found to be significantly higher among CAD patients than in healthy controls HC (0.65 vs. 0.26) (Table 3).

### 3.7. Association of ACE2 rs4240157T>C Genotypes with CAD Susceptibility Utilizing Multivariate Analysis

A multivariate analysis based on a logistic regression like odds ratio (OD) or risk ratio (RR) with 95% confidence intervals (CI) were calculated for each group to estimate the association between ACE2 rs4240157T>C genotypes and the risk of CAD—and the data are summarized in Table 7. Our results indicated that in the codominant model, the ACE2 rs4240157 CT genotype was strongly associated with an increased CAD susceptibility with an OR of 3.48, (95%) CI = (1.95 to 6.20), and *p* < 0.0001. Similarly, the ACE2 rs4240157 CC genotype was strongly associated with an increased CAD susceptibility with an OR of 14.82, (95%) CI = (7.3176 to 30.0233), and *p* < 0.0001. There is a strong association observed between the ACE2 rs4240157 ACE2-(CT+CC) genotype in the dominant inheritance model with an OR of 6.09, (95%) CI = (3.6030 to 10.3187), and *p* < 0.0001, whereas a strong association was observed between the ACE2 rs4240157 ACE2-(TT+CT) genotype in the recessive inheritance model, which leads to an increased CAD susceptibility with an OR = 7.78, (95%) CI (4.1757 to 14.4961), and *p* < 0.0001 (Table 7). In allelic comparison, the T allele was strongly associated with CAD susceptibility with an OR of 5.10, (95% CI) (3.6004 to 7.2395), and a *p*-value = 0.0001.

### 3.8. Association of Clinical Features of Coronary Artery Disease Patients with ACE2 rs4240157T>C Genotypes

**Association with gender and age:** Our results indicated that there was a significant correlation between ACE2 rs4240157T>C genotypes and the age of the CAD patients (*p* < 0.022). However, the higher incidence of heterozygosity was reported among coronary artery disease patients with younger patients (lesser than 50 years of age). (Table 8)

Association **with gender:** Moreover, the result showed that there is no association between ACE2 rs4240157T>C genotypes and the gender of patients with a (*p* < 0.33).

### 3.9. Association with Lipid Profile Biomarkers

**Association with total Cholesterol (mg/dL):** As depicted in Table 8, the statistical analysis of the correlation between ACE2 rs4240157 genotypes and Cholesterol (mg/dL) levels in the blood of coronary artery disease patients revealed a significant association (*p* < 0.0023). (Table 8)
**Association with LDL-C (mg/dL):** Moreover, the result showed that there is no association between ACE2 rs4240157T>C genotypes and LDL-C (mg/dL) levels in the blood of CAD patients with a (*p* < 0.58). (Table 8)
**Association with HDL-C (mg/dL):** The statistical analysis of the association between ACE2 rs4240157 genotypes and HDL-C (mg/dL) levels in the blood of CAD patients revealed a significant association (*p* < 0.0007). (Table 8)
**Association with Triglycerides (mg/dL)**: A significant association was observed in the triglycerides (mg/dL) between ACE2 rs4240157 genotypes and HDL-C (mg/dL) levels in the blood of CAD patients (*p* < 0.043).
**Association with Creatinine (mg/dL):** The statistical analysis of the association between ACE2 rs4240157 genotypes and Creatinine (mg/dL) in coronary artery disease patients revealed a significant association (*p* < 0.0043). (Table 8)
**Association with C-reactive protein (mg/L):** The statistical analysis of the association between ACE2 rs4240157 genotypes and C-reactive protein (mg/L) in coronary artery disease patients revealed a significant association (*p* < 0.017).
**Association with hypertension and diabetes:** Our results revealed that there was a significant association between ACE2 rs4240157T>C genotypes and hypertension (*p* < 0.049). Similarly, a significant association was reported between ACE2 rs4240157T>C genotypes and diabetes (*p* < 0.037). (Table 8)
**Association with smoking and obesity:** Our results revealed that there was no significant association between ACE2 rs4240157T>C genotypes and smoking (*p* < 0.77). Similarly, a significant association was reported between ACE2 rs4240157T>C genotypes and obesity (*p* < 0.0005).

**Table 8:** Association of clinical and biochemical features of coronary artery disease with ACE2 rs4240157T>C genotypes.

Clinical and Biochemical Features	TT	CT	CC	χ^2^	DF	*p* Value
Association with gender						
Male (96)	6	45	45	1.32	2	0.51
Female (53)	3	20	30			
Association with age						
≤50 (108)	5	55	48	8.8	2	0.012
>50 (41)	4	10	27			
Association with total Cholesterol (mg/dL)						
Cholesterol ≤200 mg (72)	5	20	47	12.11	2	0.0023
Cholesterol >200 mg (77)	4	43	30			
Association with LDL-C (mg/dL)						
LDL ≤100 mg (86)	4	38	44	1.08	2	0.58
LDL >100 mg (63)	5	30	28			
Association with HDL-C (mg/dL)						
HDL ≤40 mg (67)	4	20	43	9.93	2	0.007
HDL >40 mg (82)	5	45	32			
Association with Triglycerides (mg/dL)						
TGL ≤ 150 mg (91)	5	34	52	6.29	2	0.043
TGL > 150 mg (58)	4	31	23			
Association with Creatinine (mg/dL)						
<1.35 mg/dL(86)	3	30	53	10.91	2	0.0043
>1.35 mg/dL(63)	6	35	22			
Association with C-reactive protein (mg/L)						
<10 mg/L (65)	4	20	41	8.09	2	0.017
>10 mg/L (84)	5	45	34			
Association with hypertension						
Hyper (61)	6	20	35	6.26	2	0.049
No Hyper (88)	3	45	40			
Association with Diabetes						
T2D (75)	5	25	45	6.57	2	0.037
T2D (74)	4	40	30			
Association with Smoking						
Smoking (Yes) 82	4	37	41	0.5	2	0.77
Smoking (N0) 67	5	28	34			
Association with Obesity						
Obesity (72)	7	20	45	15.24	2	0.0005
Obesity (77)	2	45	30			
Association with Myocardial infarction (MI)						
(MI) (84)	6	35	43	0.58	2	0.748
(MI) (65)	3	30	32			

## 4. Discussion

Systemic arterial hypertension (SAH) is a leading cause of cardiovascular diseases (CVD), reaching a prevalence of 30% in the world population. As a multifactorial condition, the environmental factors and emerging weight of genetic factors need to be considered for years to come in order to tackle this disease. As well, with the application of genetic risk scores—and their continuing development, especially given that genetics can be responsible for about 40% of the inter-individual variance in blood pressure levels—will be useful. In this sense, several research groups are dedicated to studying the genetic basis of hypertension, but it has been pointed out that the small contributions of each one of the genetic factors and the interaction among them is a problem in identifying the genetic markers of the disease. It is of great importance to discuss how the *ACE* gene presents an enormous quantity of polymorphisms, with a further 77 variant sites being already identified, and, particularly in the region surrounding the *Alu* sequence (intron 16), there are 17 SNPs that have been linked with a high degree of disequilibrium in the *ACE* I/D polymorphism. Taken together, those findings allowed us to raise the hypothesis that the specific combinations (haplotypes) formed with the I and D alleles can be responsible for the associations verified in this and other studies involving the *ACE* I/D polymorphism, which can function as a genetic marker for the variation flanking it.

### 4.1. Correlation of ACE2 I/D Gene Variability with CAD Patients

The RAS (renin–angiotensin system) plays an important role in the pathophysiology of CVD (cardiovascular disease), and RAS blockade is an important therapeutic strategy in the management of CVD. The ACE polymorphism (rs4646994) is due to the presence of the insertion (I) allele or absence (deletion) of the (D) allele with a 287 bp Alu repeat sequence within intron 16, which results in three genotypes: DD, II, and ID [26]. Many studies, including a meta-analysis, have reported an association between the DD genotype and an increased risk of developing coronary artery disease [27,28,29]. However, some studies failed to confirm these findings [30,31,32,33]. The ACE gene is located on chromosome 17q23. It spans 21 kb and consists of 26 exons and 25 introns. The ACE I/D polymorphism (rs4646994) is characterized by the presence (I) or absence (D) of a 287 bp Alu repeat sequence within intron 16, resulting in three genotypes: DD, II, and ID. The frequency of the ACE (rs4646994) D allele was found to be significantly higher in CAD patients (55.55%) than in the control group (39.49%). Similarly, a higher frequency of heterozygosity was reported in CAD patients than the healthy controls (DI 61.53% vs. 31.09%). Recent findings, as well as this research here within, have shown an association between the ACE D allele, diabetes, and the risk of developing cardiovascular disease [34]. However, some studies failed to discern any relationship between the ACE gene polymorphism and CAD in patients with diabetes [35,36]. A possible mechanism suggests that the ACE DD genotype is involved in increasing the vasoconstrictor angiotensin II as well as the degradation of the vasodilator bradykinin, which increases resistance to insulin [37]. Our results are consistent with data obtained from different studies [38,39,40]; we found that the ACE (rs4646994) D allele increases the risk of CAD in T2DM patients. In contrast, several investigators were not able to find an association between the ACE D allele and the risk of CAD in the populations that they had investigated [34,41]. It is conceivable that a gene–environment interaction may play a significant role in the predisposition to CAD [35,36]. The insertion/deletion (I/D) (rs4646994) polymorphism in intron 16 of the ACE gene is considered an important genetic determinant of CAD and hypertension [37,42]. There is controversy related to the role of the ACE D/I polymorphism in the risk of CAD and myocardial infarction (MI) [43].

We reported that ACE2–D was associated with an increased susceptibility to CAD with an odds ratio that is 3.45-fold over those without the ID genotype, and also ACE2–ID was associated with increased odds for CAD by more than 2.45-fold over those without the DD genotype. In our logistic model, three independent variables were associated with the occurrence of coronary atherosclerosis—DD genotypes, hypertension, diabetes, and dyslipidemia. The logistic regression analysis confirmed that the ACE DD genotype is an important risk factor that would increase the odds for CAD by more than 8-fold over those without the DD genotype while holding other risk factors constant. Furthermore, the DD genotype and the D allele, which are concurrent with hypertension, seem to promote the risk of CAD. This result is consistent with previous reports; Kato et al [44] reported that the DD genotype of the ACE I/D polymorphism is a major risk factor for cerebral and cardiovascular events such as CAD and stroke in Japanese patients with hypertension [38]. Other studies also reported strong associations between the DD genotype and the risk of hypertension in their study populations [39]. Studies noted that smoking and the D allele increase the superoxide anion formation and degradation of the nitric oxide that cause endothelial dysfunction favoring the occurrence of coronary disease [45]. Significant differences in the genotype distribution were detected only in those children with 2 or more affected grandparents This research is suggestive that a family history of CAD and ACE I/D genotype are bidirectionally linked, whereas recent data has shown that the DD genotype and a family history of CAD are independent risk factors for CAD, and, further, that the DD genotype may increase the risk to those who also share a family history of CAD [46].

Our results indicated that there was a significant correlation between ACE2-rs4646994 I/D genotypes and the age of the coronary artery disease patients (*p* < 0.022, Table 5). However, the higher incidence of heterozygosity was reported among coronary artery disease patients who were younger than 50 years old with MI, which suggests that the contribution of genetics as a risk factor for MI occurrence became stronger among younger individuals—having none of the traditional risk factors, such as age. Similar results were reported by MM et al. [46], which showed a significant correlation of ACE2-I/D genotypes with respect to age (*p* < 0.035). On the other hand, there was a study done by Maria-Cristina Vladeanu et al. [47] that reported no statistically significant differences. Moreover, the results showed that there is no association between ACE2-rs4646994 I/D genotypes and the gender of patients with a (*p* < 0.33), and the majority of them were males. Similar results were reported by Wiam Hmimech et al. in myocardial infarction susceptibility among young Moroccan patients [48]. 

As depicted in Table 5, the statistical analysis of the correlation between ACE2-rs4646994 I/D genotypes and Cholesterol (mg/dL) levels in the blood of coronary artery disease patients revealed a significant association (*p* < 0.002). Our results are in disagreement with the results of Šeruga M, et al. [49] in Slovenia, whereas they agree with another study conducted in Egypt by Ibrahim et al. [10]. Our study revealed that that there was slightly insignificant association between ACE2-rs4646994 I/D genotypes and LDL-C (mg/dL) in CAD patients (*p* > 0.08). On the other hand, there was a significant association with HDL-C (mg/dL) in CAD patients (*p* > 0.035). This was in agreement with a study conducted in patients with coronary artery disease in the Portuguese population in 2008 by Ana I Freitas et al. [50]. Similarly, a significant correlation was reported between ACE2-rs4646994 I/D genotypes and diabetes (*p* < 0.0001). Similar results were reported for ACE2-rs4646994 I/D genotypes in T2D by Denise S. et al. [51] in Brazil. Moreover, the result agreed with another study done by Sonali Pechlivanis et al. [52] Also, our results suggest that family history, smoking, diabetes, hypertension, obesity, and the ACE DD genotype were independent risk factors for premature CAD.

### 4.2. Association of ACE2 rs4240157T>C

The amplification refractory mutation system (ARMS) is a powerful technique for detecting any mutation involving a single base change. However, the optimization step required hard work and was very time-consuming. We made small changes in the reagent concentrations, which significantly affected the PCR—especially MgCl_2_. Balancing the inner primers band was a key step. In order to balance the inner primers band, it was important to observe the intensity and which was the weakest band in order to promote this band by increasing its concentration. Gradient PCR was performed using different annealing temperatures and a lesser number of cycles were used (25 to 30 cycles). This optimization was achieved in a series of optimization experiments in a single run by the gradient PCR machine following some previous reported studies [22,23,24,25]. The annealing temperature via gradient PCR was optimized from 58 °C to 62 °C, but the best results were obtained at a temperature 58 °C. The use of tetra-primer ARMS-PCR meets the expectations of modern genomic research and allows the study of ACE2 rs4240157T>C in a fast, reliable, and low-cost way.

Patel SK et al. [53] reported among persons of Australian descent that ACE2 rs4240157T>C exhibited a higher T2D with a risk of hypertension, rs1978124 was associated with a risk of T2D related left ventricular remodeling. ACE2 rs4240157T>C was correlated with two types of dyslipidemia (TRIG and HDL-C, both *p* < 0.05). Liu C et al. [54] also found that the levels of HbA1C in T2D Uygur patients with high diabetic risk genotypes at three loci (rs2074192, rs4240157, and rs879922) increased significantly, suggesting that those would have a potentially higher risk of cardiovascular events.

Wang YX et al. [55] found that rs4646188 was associated with a high risk of systolic blood pressure (SBP ≥ 130 mmHg) and moderate risk of diastolic blood pressure (DBP ≥ 80 mmHg). SNP rs4240157 was only associated with a high risk of increased SBP, while rs2074192 was only associated with a moderate risk of increased diastolic blood pressure DBP. Harold E Bays et al. [56] showed that there was a 1.5–2.0-fold increase in the risk of cardiovascular events when systolic blood pressure (SBP) increased from 130 mmHg to 139 mmHg. These results suggest that those elevated SBP risk-related loci may be genetic factors contributing to T2D-related cardiovascular events risk in Uygurs. ACE2 SNP rs4240157 were associated with high TRIG level, and almost all diabetic risk related to ACE2 SNPs was associated with low HDL-C levels. The ACE2 rs4240157 C allele (CT+CT or CC+CG) of rs4240157 was associated with essential hypertension (EH) (*p* = 0.012).The findings of Pan Y et al. [57] are consistent with the observations made by Patel et al. [53], who reported ACE2 SNPs (rs2074192, rs4240157, and rs4646188) were associated with a higher risk of hypertension. Pan Y et al. [57] reported four ACE2 SNPs (rs2074192, rs4240157, rs4830542, and rs879922) that were associated with hypertension and increased triglycerides, but only rs4240157 also exhibited an association with a high risk of stroke. In men, hypertension was more prevalent with the *ACE2* rs2074192 C allele (*p* = 0.023) and rs4240157 G allele (*p* = 0.016) [57]. There was a significant association in the *ACE2* rs4240157 SNP with a higher Left Ventricular Mass.

## 5. Conclusions

It was concluded that the ARMS technique and MS-PCR assay proved to be fast, accurate, and reliable for ACE2-rs4240157T>C and ACE1-rs4646996 D>I, respectively, and can be used as a potential molecular tool in the diagnosis of genetic diseases in undeveloped and developing countries—where there might be a shortage of medical resources and supplies. ACE1-I>D genotypes were strongly associated with T2D, hypertension, and obesity (*p* < 0.002). Besides the ACE2-rs4240157 CT heterozygosity genotype, the T allele was strongly associated with CAD susceptibility. Future longitudinal studies in different ethnic populations with larger sample sizes are recommended to validate these findings.

## Figures and Tables

**Table 1:** Primer sequences for ACE2 gene polymorphisms.

Direction	Primer Sequence	PCR Product	Annealing Temperature
**T-ARMS-PCR primers for ACE-1-rs4646994 I/D gene polymorphism**			
Fo primer D/I	5′-GAGGCTGAGATGGAAGGATTG-3′	488 *bp*	Touch down PCR
Ro primer D/I	5′-GCTCTCCCAACACCACATTAC-3′	712 *bp*	69 to 69 °C
FI primer A	5′-TCTGACGAATGTGATGGCCCCA-3′	271 *bp*	
RI primer G	5′-AACAGGTCTTCATATTTCCGGTAC-3′	200 *bp*	
**T-ARMS-PCR primers for ACE2 rs4240157T>C gene polymorphism**			
ACE2 Fo	GCTGAGTTCTCAAAATAATGCCATAGAT	386 bp	60 °C
ACE2 Ro	GCATTTCTTTCCAATCATTAAGAGTTCA		
ACE2 FI-T	GCCTCAGAACATTACAGAATCAACCT	244 bp	
ACE2 RI-C	GAGGGTTGGTAAATAGTGTTCAGTGG	194 bp	
**Mutation specific *PCR* primers for ACE-1-rs4646994 I/D gene polymorphism**			
ACE-F	5′-CTGGAGACCACTCCCATCCTTTCT-3′	490-bp (II)	58 °C
ACE-R	5′-GATGTGGCCATCACATTCGTCAGAT-3′.	190-bp (DD)	

**Table 2:** Demographic features of biochemical parameters of coronary artery disease patients.

Parameters	Value (%)	
**CAD Patients**	**150**	**%**
Male	96	64%
Female	54	36%
Age < 50	108	72%
Age > 50	42	28%
Cholesterol ≤ 200 (mg/dL)	72	48%
Cholesterol > 200 (mg/dL)	78	52%
LDL ≤ 100 (mg/dL)	86	57.33%
LDL > 100 (mg/dL)	64	42.66%
HDL ≤ 40 (mg/dL)	67	44.66%
HDL > 40 (mg/dL)	83	55.33%
TGL ≤ 150 (mg/dL)	91	60.66%
TGL > 150 (mg/dL)	59	39.33%
Creatinine < 1.35 mg/dL	86	57.33%
Creatinine > 1.35 mg/dL	64	42.66%
C-reactive protein < 10 mg/L	65	43.33%
C-reactive protein > 10 mg/L	85	56.66%
Hypertension	61	40.66%
No hypertension	89	59.33%
T2D	76	50.66%
No T2D	74	49.33%
Smoking (Yes)	82	54.66%
Smoking (No)	68	45.33%
Obesity	72	48%
No Obesity	78	52%
Myocardial infarction (MI)	84	56%
No Myocardial infarction (MI)	66	44%

**Table 3:** Clinical association of ACE2 I/D gene variation between coronary artery disease cases and controls.

Subjects	n=	II	DI	DD	Df	χ^2^	I	D	*p* Value
Cases	149	09(6.0%)	65(43.62%)	75(50.33%)	2	9.46	0.28	0.72	0.008
Controls	150	23(15.33%)	70(46.66%)	55(36.66%)			0.40	0.60	

**Table 4:** Multivariate analysis of ACE2 I/D polymorphism in the coronary artery disease patients and healthy controls.

Genotypes	Healthy Controls	CAD Cases	OR (95% CI)	Risk Ratio(RR)	*p*-Value
	(n = 148)	(n = 149)			
Codominant inheritance model					
ACE2–II	23	09	1 (ref.)	1 (ref.)	
ACE2–ID	70	65	2.37 (1.0231 to 5.5041)	1.38 (1.0572 to 1.8175)	0.044
ACE2–DD	55	75	3.48 (1.4961 to 8.1170)	1.69 (1.2643 to 2.2827)	0.003
Dominant inheritance model					
ACE2–II	23	09	1 (ref.)	1 (ref.)	
ACE2–(ID+DD)	125	140	2.86 (1.2765 to 6.4179)	1.52 (1.1850 to 1.9593)	0.010
Recessive inheritance model					
ACE2–(II+ID)	93	74	1 (ref.)	1 (ref.)	
ACE2–DD	55	75	1.71 (1.0787 to 2.7226)	1.31 (1.0333 to 1.6768)	0.022
Allele					
ACE2–I	116	83	1 (ref.)	1 (ref.)	
ACE2–D	180	215	1.66 (1.1830 to 2.3557)	1.27 (1.0906 to 1.5003)	0.003

**Table 5:** Association of biochemical parameters and genotype distribution of ACE2 I/D genotypes and coronary artery disease patients.

Clinical Feature	II	DI	DD	χ^2^	DF	*p* Value
Association with gender						
Male (100)	5	45	50	2.18	2	0.33
Female (49)	4	20	25			
Association with age						
≤50 (108)	3	50	55	7.9	2	0.022
>50 (41)	6	15	20			
Association with total Cholesterol (mg/dL)						
Cholesterol ≤ 200 mg (72)	6	21	45	11.98	2	0.002
Cholesterol > 200 mg (77)	3	44	30			
Association with LDL-C (mg/dL)						
LDL ≤ 100 mg (86)	4	32	50	5.03	2	0.080
LDL > 100 mg (63)	5	33	25			
Association with HDL-C (mg/dL)						
HDL ≤ 40 mg (67)	3	37	27	6.68	2	0.035
HDL > 40 mg (82)	6	28	48			
Association with Triglycerides (mg/dL)						
TGL ≤ 150 mg (91)	6	30	55	10.95	2	0.004
TGL > 150 mg (58)	3	35	20			
Association with Creatinine (mg/dL)						
<1.35 mg/dL(86)	6	30	50	6.32	2	0.42
>1.35 mg/dL(63)	3	35	25			
Association with C-reactive protein(mg/L)						
<10 mg/L (65)	5	15	45	19.36	2	0.0001
>10 mg/L (84)	4	50	30			
Association with hypertension						
Hyper (61)	3	37	21	12.28	2	0.002
No Hyper (88)	6	28	54			
Association with Diabetes						
T2D (75)	4	47	24	22.7	2	0.0001
T2D (74)	5	18	51			
Correlation with Smoking						
Smoking (Yes)	6	36	40	0.58	2	0.74
Smoking (N0)	3	29	35			
Association with Obesity						
Obesity (72)	7	25	40	6.41	2	0.040
Obesity (77)	2	40	35			
Association with Myocardial infarction (MI)						
(MI) (84)	5	24	55	18.77	2	0.0001
(MI) (65)	4	41	20			

**Table 6:** Statistical comparisons between CAD patients’ ACE2 rs4240157T>C gene.

Subjects	n =	TT%	CT%	CC%	Df	χ^2^	T	C	*p* Value
Cases	150	27(18%)	54(36%)	69(46%)	2	66.44	0.35	0.65	0.0001
Controls	152	87(57.23%)	50(31.57%)	15(9.86%)			0.74	0.26	

**Table 7:** Multivariate analysis of ACE2 rs4240157T>C gene polymorphism with coronary artery disease patients.

Genotypes	Healthy Controls (n = 152)	CAD Cases (n = 150)	OR (95% CI)	Risk Ratio (RR)	*p* Value
**Codominant**					
ACE2-TT	87	27	1 (ref.)	1 (ref.)	
ACE2-CT	50	54	3.48 (1.95 to 6.20)	1.58 (1.2683 to 1.986)	0.0001
ACE2-CC	15	69	14.82 (7.3176 to 30.0233)	4.27 (2.6713 to 6.8374)	0.0001
**Dominant**					
ACE2-TT	87	27	1 (ref.)	1 (ref.)	
ACE2-(CT+CC)	65	123	6.09 (3.6030 to 10.3187)	2.20 (1.7685 to 2.7550)	0.0001
**Recessive**					
ACE2-(TT+CT)	137	81	1 (ref.)	1 (ref.)	
ACE2-CC	15	69	7.78 (4.1757 to 14.4961)	3.51 (2.1998 to 5.6302)	0.0001
**Allele**					
ACE2-T	224	108	1 (ref.)	1 (ref.)	
ACE2-C	78	192	5.10 (3.6004 to 7.2395)	2.33 (1.9093 to 2.8569)	0.0001

## Data Availability

All the data associated with the current study has been presented in this manuscript.
